# Cultured Bacteria in the Abdominal Wall Incision During the Realisation of Caesarean Section in Cows: A Preliminary Study

**DOI:** 10.3390/vetsci12020098

**Published:** 2025-01-30

**Authors:** Salem Djebala, Elise Coria, Florian Munaut, Linde Gille, Justine Eppe, Nassim Moula

**Affiliations:** 1 Farm Animal Clinic, School of Veterinary Medicine, University College of Dublin, Belfield, Dublin 4, D04W6F6 Dublin, Ireland; 2 Clinical Department of Ruminants, University of Liège, Quartier Vallée 2, Avenue de Cureghem 7A-7D, 4000 Liège, Belgium; elise.coria@hotmail.com (E.C.); fmunaut@hotmail.com (F.M.); linde.gille@msd.com (L.G.); justine.eppe@uliege.be (J.E.); 3 MSD Animal Health Benelux, 1170 Watermael-Boitsfort, Belgium; 4 Department of Animal Production, University of Liège, Quartier Vallée 2, Avenue de Cureghem 6, 4000 Liège, Belgium; 5 GIGA—Animal Facilities—ULiège—B 34, 4000 Liège, Belgium

**Keywords:** caesarean section, bacterial culture, abdominal incision contamination, wound infection

## Abstract

Although caesarean section (CS) is a common surgery, the bacteria contaminating the abdominal incision during this operation are not documented. This study aimed to enhance perioperative antibiotic therapy and reduce post-operative surgical site infections by describing the bacteria contaminating the abdominal incision during CS. Bacterial culture was performed on swabs sampled from the abdominal incision (muscular layers) of 23 cows during the realisation of CS. Bacteriology was positive in 6/22 samples. while 16/22 were negative (*p* = 0.03), and one sample was contaminated. All identified bacteria were aerobic, and 8/14 isolates were Gram-positive and 6/14 were Gram-negative (*p* = 0.7). The most encountered species were *Acinetobacter* spp., *Aerococcus viridans*, *Neisseria* spp. and *Streptococcus* spp.; other species were isolated only once. The abdominal incision of elective CS can be contaminated by ubiquitous aerobic Gram-positive and Gram-negative species coming from the environment. This contamination might be avoided by using a better aseptic protocol and by improving the conditions in which CSs are performed. However, more samples are required, and antibiotic susceptibility determination is needed to identify the adequate pre-operative antibiotic.

## Simple Summary

Caesarean section (CS) is the most common operation performed in cattle. This surgery is often performed in a contaminated environment, increasing the risk of post-operative surgical wound infection. For this reason, veterinarians administer antibiotics to reduce the occurrence of these complications. To improve the efficacy of injected antibiotics, this study aimed to describe the bacteria likely to be encountered in the abdominal wall incision during a CS. Swabs were taken from the abdominal incision of 23 cows during the procedure and sent to the laboratory for bacterial culture. The results showed that no bacteria were cultured in the majority of samples (16/22), while 14 isolates were observed in 6/22 swabs and one sample was considered contaminated. The identified bacteria were aerobic, and some of them (6/14) were Gram-negative and others (8/14) were Gram-positive bacteria. The main identified species were *Acinetobacter* spp., *Aerococcus viridans*, *Neisseria* spp. and *Streptococcus* spp. In conclusion, the abdominal wound incision of CS is frequently contaminated by environmental bacteria. This contamination might be avoided by an increased focus on working aseptically and by improving the general conditions in which CS are performed.

## 1. Introduction

Caesarean section (CS) is a common surgery in Belgium [1,2,3,4]. Although Belgian vets are experienced in the realisation of CS [5,6], post-operative complications account for 70% of insurance claims [7,8]. Indeed, CS are generally performed in barns with limited facilities [9], increasing the likelihood of surgical site contamination [1,10,11] and the occurrence of post-operative complications [8,10,12,13].

To prevent complications, vets administrate antibiotics [2,3,5,6,14]. However, following the lack of clear guidelines, practitioners refer to their own experience and non-evidence statements to design the antibiotic protocol [2,3,14]. In fact, around 40% of surveyed vets apply an antibiotic between the muscular layers of the surgical incision in addition to the antibiotic administered through the muscle and peritoneum. Consequently, a large amount of antibiotics is injected during CS [5,6], increasing the risk of bacterial resistance occurrence [15,16].

To implement an adequate pre-operative antibiotic therapy, the bacteria contaminating the surgical site of CS should be known. Until now, few studies have been conducted with this purpose. A first study described the bacteria cultured in 23 foetal fluids sampled during CS. The identified strains were anaerobic Gram-positive, coming from the vaginal contamination of foetal fluids [1]. A second study highlighted the bacteria cultured in the peritoneum of 76 cows during CS. The identified bacteria were aerobic and Gram-negative due to an environmental contamination [10,11].

These studies brought clear insights about the bacteria contaminating the uterus and the peritoneum during CS [1,10,11]. However, there are no data regarding the bacteria contaminating the abdominal wall incision, although this spot might be highly contaminated since abdominal wound infection is the most common complication of CS [3,7].

As such, the current study aimed to describe the bacteria contaminating the abdominal wall incision. Furthermore, it is intended to aid in the selection of the adequate antibiotic therapy to reduce post-operative complications and antimicrobial consumption.

## 2. Materials and Methods

All procedures received the approval of the Ethical Committee of Liège University (Number: 2142).

### 2.1. Animal Description and CS Realisation

Samples were collected during CS realisation from 23 healthy Belgian Blue cows that had not received any treatment for seven months beforehand. All CS were performed before full cervical dilatation on cows with an alive foetus and intact foetal membranes [17,18]. The CS were performed in the cattle barn, a highly contaminated environment, by a single experienced veterinarian following the procedure described by Djebala et al. [10,11].

### 2.2. Samples Collection

Samples were taken after replacing the sutured uterus in the abdominal cavity and before starting the abdominal wall sutures. A swab (STERILER^®^, Piove di Sacco, Italy) was swiped over a line of 10 cm, on both sides of the ventral commissure of the abdominal wall incision, on the internal oblique muscle. The sample was kept at 4 °C and dispatched rapidly to the laboratory to perform a bacterial culture.

### 2.3. Bacterial Culture and Laboratory Analysis

The samples were used for aerobic and anaerobic bacteriological culture. Samples for aerobic culture were grown on Columbia agar, Gassner and Columbia/Nalidixic acid agar media (Thermo Fisher Scientific, Brussels, Belgium) at 37 ± 2 °C. Samples for anaerobic culture were grown under anaerobic conditions on Schaedler medium (Thermo Fisher Scientific, Brussels, Belgium) at 37 ± 2 °C. Two readings were performed after 18–24 h and 36–48 h of incubation. Bacterial identification of positive culture was performed by the Maldi Biotyper^®^ (Bruker Daltonics, Bremen, Germany) following standard protocol. The culture was considered “negative” if no bacterial growth was observed and “positive” when bacteria were cultured.

### 2.4. Statistical Analysis

Statistical analyses were performed using SAS (SAS/STAT^®^ User’s Guide, Version 8.2. Cary, NC, USA: SAS Institute Inc., 2001). Descriptive analysis was achieved for the number of samples taken in each farm and the number of bacteria identified in each positive sample.

Data distribution was verified with a Shapiro–Wilk test, and the median was used to display non-normal distributed results.

Chi-square and Fisher tests were used for comparison between the number of positive and negative samples, the number of samples showing one isolate and those showing two or more, the number of Gram-positive and Gram-negative species and isolates, the number of Gram-positive aerobic and Gram-negative aerobic species and isolates and the frequencies of the identified species.

The procedure “Proc-Freq” in SAS was used for all statistical analyses; the cut-off of significance was *p* < 0.05.

## 3. Results

In total, 23 cows coming from 14 farms were sampled during CS realisation. One to three samples were taken from each farm with a median of 1.5 samples.

Bacteriology was negative in the majority of CS (16/22; 72.72%); it was positive in only 6/22 (27.27%) samples (*p* = 0.03). One sample was considered to be contaminated since a multitude of non-characterised species grew in it. The number of bacterial species identified in the positive samples varied between one and four, with a median of two species. Among the six positive samples, one isolate was found in two samples, while two or more isolates were found in the other positive samples (*p* = 0.56).

**Figure 1:**
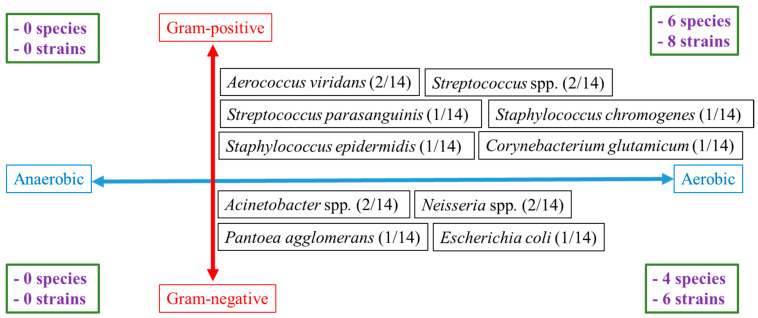
Features (growing in aerobic vs. strict anaerobic environment and Gram-positive vs. Gram-negative) of the bacteria cultured in the positive samples (6/22) [19].

In total, 14 isolates belonging to 10 species were identified. All cultured bacteria were aerobic. Nevertheless, 6/10 of identified species were Gram-positive and 4/10 were Gram-negative (*p* = 0.65). Among the 14 identified isolates, eight were Gram-positive and six were Gram-negative (*p* = 0.7). The most encountered bacterial species were *Acinetobacter* spp. (2/14), *Aerococcus viridans* (2/14), *Neisseria* spp. (2/14) and *Streptococcus* spp. (2/14); the other species were only identified once (*p* = 0.9) (Figure 1, Table 1).

## 4. Discussion

To the best of our knowledge, this research is the first study describing the bacterial contamination of the abdominal wall incision during the realisation of CS. Wound infections are the most frequent post-operative complication of CS [8,12,13,20,21,22], and abdominal incision is the gateway for the environmental bacteria contaminating the peritoneum [10,11]. Accordingly, the outcome of this research will provide more insights to improve pre-operative antibiotic therapy and to prevent post-operative complications.

The swabs were sampled in the ventral part of the abdominal incision to increase the likelihood to find positive samples. Indeed, this spot is supposed to be the most contaminated area of the abdominal incision since it is well exposed to the environmental bacteria, foetal fluid contamination and surgeon manipulations [2,3,14,21].

Although CS were performed in field conditions in a highly contaminated environment, the majority of samples showed a negative culture [10]. This may reflect an underestimation due to the limitations of bacterial culture compared to other identification methods [11,23]. However, several isolates were identified in positive samples. This might be subsequent to antisepsis disruption and environmental contamination, though the surgeon did not mention any trouble during these CSs. This hypothesis is supported by the ubiquitous features of the identified species [19], assuming the environmental contamination of the surgical site [10]. However, this finding could also result from a possible contamination of the swabs during sampling [24,25].

Although the used swabs contained an agar medium to protect the anaerobic bacteria [26], no strict anaerobic species were identified. This finding discards the assumption of the abdominal incision contamination by the foetal fluids containing anaerobic species [1]. Indeed, in this research, CSs were carried out before the foetal fluids’ bag tearing [10,11,17,18], preventing the spread of the vaginal flora to the foetal fluids and the surgical site. Nevertheless, the absence of strict anaerobic isolates could be related to the difficulties faced in culturing anaerobic species [10,11,26].

In contrast to the previous reports where Gram-positive bacteria were mainly identified in the foetal fluids [1] and Gram-negative were the main contaminant of the peritoneum [10,11], in this research, no statistical difference was noticed between the number of Gram-positive and Gram-negative isolates found in the abdominal incision of CS. Therefore, if the goal of a prophylactic antibiotic is to target the dominant bacterial population encountered during surgery [10,11,27,28,29], this research did not highlight the species that should have been targeted by the preventive antibiotic. Penicillin is the molecule advised by the Belgian expertise centre of Antimicrobial Consumption and Resistance in Animals (AMCRA) for CS [30]. This recommendation might fit with the outcomes of this study, since penicillin targets Gram-positive bacteria [31]. Accordingly, the administration of penicillin should reduce the bacterial charge of the abdominal incision by more than 50% during CS [30]. Moreover, antibiotics mainly targeting Gram-negative bacteria are discouraged for use as preventive treatment in Belgium [32].

This study could have brought more recommendations concerning the molecule that should be used during CS, if antimicrobial susceptibility testing was performed for the identified bacteria. Furthermore, more samples are required to draw stronger conclusions about the kind of bacteria contaminating the abdominal incision. This study might be considered a pioneer study alongside the research of Mijten et al. [1] and Djebala et al. [10,11]. However, given the different conclusions of these studies, better knowledge of the bacteria contaminating the surgical site requires a better designed study. Indeed, samples should be taken from the uterus, the peritoneum and the abdominal incision from each involved cow to find the bacteria contaminating the whole surgical site of the CS.

## 5. Conclusions

CS is a clean contaminated surgery, since most of the samples are bacteriologically negative. The abdominal wall incision is contaminated by aerobic Gram-positive and Gram-negative species likely coming from the environment.

## Figures and Tables

**Table 1:** This bacterial culture’s results for each sampled cow and farm: 6/23 positive samples, 1/23 contaminated sample and 16/23 negative samples.

Origin of the Sample (Farm)	Identification of Samples	Number of Species in Each Sample	Bacterium 1	Bacterium 2	Bacterium 3	Bacterium 4
**A** (1/2 positive)	1	0	*/*	*/*	*/*	*/*
	2	2	*Aerococcus viridans*	*Corynebacterium glutamicum*	*/*	*/*
**B** (1/2 positive)	3	0	*/*	*/*	*/*	*/*
	4	4	*Neisseria* spp.	*Staphylococcus epidermidis*	*Streptococcus parasanguinis*	*Streptococcus* spp.
**C** (0/1 positive)	5	0	*/*	*/*	*/*	*/*
**D** (1/1 positive)	6	1	*Acinetobacter* spp.	*/*	*/*	*/*
**E** (0/1 positive)	7	0	*/*	*/*	*/*	*/*
**F** (0/1 positive)	8	0	*/*	*/*	*/*	*/*
**G** (0/2 positive)	9	0	*/*	*/*	*/*	*/*
	10	0	*/*	*/*	*/*	*/*
**H** (1/2 positive)	11	0	*/*	*/*	*/*	*/*
	12	2	*Acinetobacter* spp.	*Aerococcus viridans*	*/*	*/*
**I** (0/1 positive)	13	0	*/*	*/*	*/*	*/*
**J** (1/2 contaminated)	14	contaminated	*/*	*/*	*/*	*/*
**K** (0/2 positive)	15	0	*/*	*/*	*/*	*/*
	16	0	*/*	*/*	*/*	*/*
**L** (2/3 positives)	17	4	*Neisseria* spp.	*Pantoea agglomerans*	*Staphylococcus chromogenes*	*Streptococcus* spp.
	18	1	*Escherichia coli*	*/*	*/*	*/*
	19	0	*/*	*/*	*/*	*/*
**M** (0/3 positive)	20	0	*/*	*/*	*/*	*/*
	21	0	*/*	*/*	*/*	*/*
	22	0	*/*	*/*	*/*	*/*
**N** (0/1 positive)	23	0	*/*	*/*	*/*	*/*

## Data Availability

All data are available in the manuscript.
